# Regulatory Mechanism of Transcription Factor *AhHsf* Modulates *AhHsp70* Transcriptional Expression Enhancing Heat Tolerance in *Agasicles hygrophila* (Coleoptera: Chrysomelidae)

**DOI:** 10.3390/ijms23063210

**Published:** 2022-03-16

**Authors:** Jisu Jin, Yiran Liu, Xiaocui Liang, Yiming Pei, Fanghao Wan, Jianying Guo

**Affiliations:** 1 State Key Laboratory for Biology of Plant Diseases and Insect Pests, Institute of Plant Protection, Chinese Academy of Agricultural Sciences, Beijing 100193, China; jinjisu9110@163.com (J.J.); liuyiran0907@163.com (Y.L.); 15373606850@163.com (X.L.); 82101202330@caas.cn (Y.P.); wanfanghao@caas.cn (F.W.); 2 Agricultural Genomics Institute at Shenzhen, Chinese Academy of Agricultural Sciences, Shenzhen 518120, China

**Keywords:** *Agasicles hygrophila*, transcription factor *AhHsf*, heat shock protein 70 promoter (*Hsp70*p), cell transfection, real-time quantitative PCR (RT-qPCR), inverse PCR (I-PCR)

## Abstract

*Agasicles hygrophila* is a classical biological agent used to control alligator weed (*Alternanthera philoxeroides*). Previous research has indicated that the heat shock factor (HSF) is involved in regulating the transcriptional expression of *Hsp70* in response to heat resistance in *A. hygrophila.* However, the regulatory mechanism by which *AhHsf* regulates the expression of *AhHsp70* remains largely unknown. Here, we identified and cloned a 944 bp *AhHsp70* promoter (*AhHsp70*p) region from *A. hygrophila.* Subsequent bioinformatics analysis revealed that the *AhHsp70*p sequence contains multiple functional elements and has a common TATA box approximately 30 bp upstream of the transcription start site, with transcription commencing at a purine base approximately 137 bp upstream of ATG. Promoter deletion analyses revealed that the sequence from −944 to −744 bp was the core regulatory region. A dual-luciferase reporter assay indicated that overexpressed *AhHsf* significantly enhanced the activity of *AhHsp70*p. Furthermore, qPCR showed that *AhHsp70* expression increased with time in *Spodoptera frugiperda* (Sf9) cells, and *AhHsf* overexpression significantly upregulated *AhHsp70* expression in vitro. Characterization of the upstream regulatory mechanisms demonstrated that *AhHsf* binds to upstream *cis*-acting elements in the promoter region of *AhHsp70* from −944 to −744 bp to activate the AhHSF–AhHSP pathway at the transcriptional level to protect *A. hygrophila* from high temperature damage. Furthermore, we proposed a molecular model of *AhHsf* modulation of *AhHsp70* transcription following heat shock in *A. hygrophila*. The findings of this study suggest that enhancing the heat tolerance of *A. hygrophila* by modulating the upstream pathways of the *Hsp* family can improve the biocontrol of *A. philoxeroides*.

## 1. Introduction

Insects are poikilothermal organisms that adapt to different environmental temperatures through a variety of physiological and biochemical responses, and this ability directly influences their population expansion and distribution [1,2]. Moreover, climatic adaptability can drive insect adaptive evolution, promoting population differentiation and even the formation of new species [3,4,5].

Organisms respond to the chemical and physiological stresses associated with elevated temperatures by increasing the synthesis of heat shock proteins (Hsps) [6,7,8,9,10], which protect cells from hyperthermic stress by binding to denatured proteins and facilitating their correct refolding [8,11,12]. Under normal conditions, the expression of *Hsp* is typically maintained at very low levels, although expression levels can be rapidly upregulated in response to stress conditions such as heat shock [13,14]. In terms of insect thermotolerance, *Hsp70*, a member of the highly conserved chaperone class of proteins, is a typical representative of the heat shock gene family [15,16]. The *Hsp70* promoter has beenis used extensively in transgenic and gene therapy studies to drive the expression of exogenous genes [14,17], and numerous in vitro studies have sought to elevate its transcriptional activity for selected transgenes [18]. In this regard, the regulation of transcriptional gene expression via DNA elements such as promoters and enhancers playsan important role in controlling the expression of genes associated with stress resistance [19,20,21,22].

The heat shock response represents a typical case of inducible gene expression [15,23] that involves transcriptional activation mediated by the heat shock factor (HSF), which binds to specific elements in the heat shock gene [24,25,26,27]. Among the stress-related proteins, members of the most abundant and conserved Hsp70 family have frequently been proposed as potential biomarkers of cellular toxicity [28]. Li et al. found that *AtHsf* regulates the expression of stress-responsive genes (*Hsp*s) to enhance tolerance to heat and other abiotic stresses in *Arabidopsis* by functional analysis of *AtHsf* knockout mutants and *AtHsf* overexpressing transgenic plants [25]. In *Caenorhabditis elegans*, Baird et al. found that HSF-1 modulates the calcium-binding protein PAT-10 to increase thermotolerance and longevity during thermal stress [23]. In addition, it has been reported that post-translational modifications of Hsp70 family proteins including phosphorylation, acetylation, ubiquitination, aminoylation, and ADP ribosylation play an important role in regulating Hsp70 activity [29].

The flea beetle *Agasicles hygrophila* Selman & Vogt, which is used as an important biological control agent for alligator weed (*Alternanthera philoxeroides* [Amaranthaceae]) is markedly influenced by temperature [30,31,32]. Previous field surveys in Hunan Province, China have revealed that the population density of *A. hygrophila* decreases sharply during mid-summer from July to September, thereby limiting the control of *A. philoxeroides* population growth at this time of the year [33,34]. The correlation between high temperatures (>36 °C and even above 39 °C) and the population decline of this beetle species indicates that extremely high temperatures may be the primary factor suppressing the control of alligator weed during mid-summer [35,36,37]. In addition, temperature data for Changsha City collected from 2003 to 2013 indicated that in July and August, the frequencies of extremely high temperatures exceeding 36 °C were 42.5% and 32.1%, respectively, and the daily maximum temperatures recorded were often above 39 °C [37]. (Supplementary Table S1; [China Meteorological Data, http://data.cma.gov.cn/, accessed on 6 August 2020]). *Alternanthera philoxeroides* (Mart.) Griseb., an aquatic amaranth native to South America [38], was introduced into China in the 1930s as a forage crop [39], and has spread throughout the southern regions of the country, becoming one of the most noxious weeds in China [39,40]. This alien species has been recognized as a serious aquatic pest problem threatening aquatic ecosystems worldwide and is currently listed as one of the 16 most serious invasive species in China [31,32,41].

In our efforts to elucidate the molecular mechanisms underlying the heat resistance of *A. hygrophila,* we previously demonstrated the importance of the *AhHsp70* in the thermotolerance of this beetle [42] and subsequently isolated and identified a heat shock factor *(AhHsf*) and its putative downstream target gene, *AhHsp70*. Our findings provide evidence that *AhHsf* is involved in regulating the transcriptional expression of *AhHsp70* in response to the thermotolerance of *A. hygrophila* [43]. However, the mechanism by which *AhHsf* regulates the expression of *AhHsp70* remains poorly understood.

In the present study, we sought to verify our hypothesis that *AhHsp70* plays a pro-active role in *A. hygrophila* stress resistance by interacting with *AhHsf*. To this end, we determined the sequence of the *AhHsp70* promoter (*AhHsp70*p) using the inverse PCR (I-PCR) technique of chromosome walking, and performed subsequent bioinformatics analysis. In addition, we characterized the *AhHsp70* core promoter region by promoter deletion analysis. Finally, using a dual-luciferase reporter (DLR) assay system, we determined the interaction between *AhHsf* and *AhHsp70*p and established that *AhHsf* directly targets the *AhHsp70* core promoter region to activate the AhHSF–AhHSP70 signaling pathway by regulating the transcriptional expression of *AhHsp70*. These findings provide important insights into the regulatory mechanisms associated with the response of *A. hygrophila* to high external temperatures, and will potentially contribute to predicting the efficacy of biocontrol using this beetle in the face of ongoing climate change.

## 2. Results

### 2.1. Analysis of the AhHsp70p Sequence in A. hygrophila

**Figure 1:**
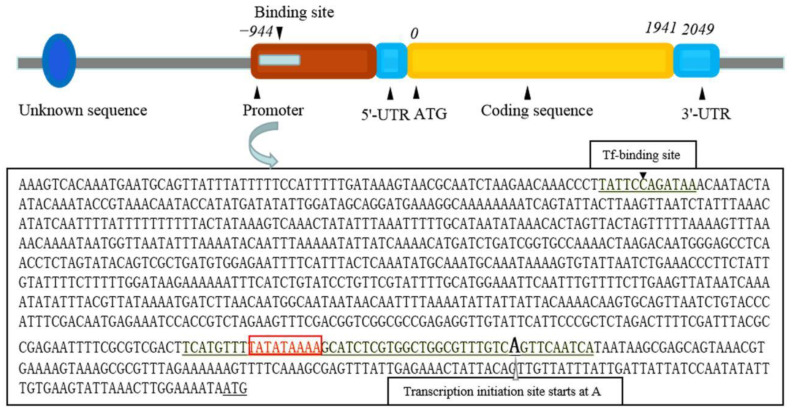
Schematic representation of the promoter sequence of the *AhHsp70* gene (*AhHsp70*p) from *Agasicles hygrophila* based on bioinformatics analysis. The *AhHsp70*p sequence contains multiple functional elements and a common TATA box at approximately 30 bp upstream of the transcription start site. Transcription is believed to commence at a purine base approximately 137 bp upstream of the coding region of ATG. The blue circle on the left represents other unknown sequences upstream of the *AhHsp70*p. −944, 0, 1941, and 2049 refer to the cloned promoter sequence location, the start codon location, the end of the coding region sequence location, and the 3′-UTR sequence location, respectively.

In this study, we isolated and identified a 944-bp upstream promoter sequence of the *AhHsp70* gene (*AhHsp70*p) from *A. hygrophila* using I-PCR which was deposited in the NCBI database (GenBank accession number: MZ351037) (Figure 1). Bioinformatics analysis revealed that the *AhHsp70*p sequence contains multiple functional elements including a common TATA box, which is a DNA sequence recognized by transcription factors, located approximately 30 bp upstream of the transcription start site. Berkeley Drosophila Genome Project: Neural network promoter prediction indicated that *AhHsp70*p transcription commences from a purine base approximately 137 bp upstream of the ATG coding region (Figure 1). Furthermore, JASPAR and Tfsitescan predictions indicated that *AhHsp70*p has transcription factor binding sites at approximately −850 bp (Figure 1), whereas MethPrimer analysis revealed that *AhHsp70*p does not contain CpG islands (Supplementary Figure S1).

### 2.2. Analysis of AhHsp70 Promoter Reporter Plasmid Activity

**Figure 2:**
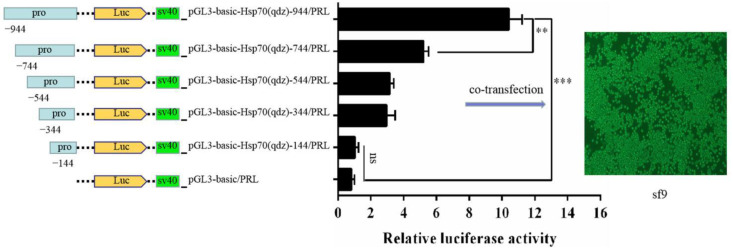
Analysis of the luciferase activity of truncated sequences of *AhHsp70*p. In this experiment, *Spodoptera frugiperda* (Sf9) cells were used for the stable expression of *AhHsp70* in vitro. Sf9 cells were transfected with recombinant plasmids containing *AhHsp70*p sequences of differing deletion length or pGL3-Basic and pRL-TK as controls for 48–96 h and the cells were harvested for the luciferase activity assay. qdz represents the *AhHsp70* promoter. All values are shown as the mean ± SD. The data were analyzed using the Student’s *t*-test. ** *p* < 0.01, extremely significant; *** *p* < 0.001, extremely extremely significant; ns, not significant.

To determine the optimal co-transfection efficiency of the recombinant plasmid (pGL3-basic-*AhHsp70*p) and internal reference plasmid (PRL-TK), we assessed different ratios of the recombinant plasmid and the internal control vector plasmid used for co-transfection and established that the optimal transfection efficiency was obtained at a ratio of 10:1 (Supplementary Figure S2, *F*_(144)_ = 48.01, *p* < 0.0001; *F*_(344)_ = 425.03, *p* < 0.0001; *F*_(544)_ = 44.49, *p* < 0.0001; *F*_(744)_ = 124.66, *p* < 0.0001; *F*_(944)_ = 133.42, *p* < 0.0001). Thereafter, we analyzed the activity of the *AhHsp70*p reporter plasmids in *Spodoptera frugiperda* *(S*f9) cells using thea *TransDetect*^®^ DLR assay system. The results indicated that, with the exception of pGL3-basic-*AhHsp70*p-144, the reporter plasmids showed significantly higher activity than the control plasmid (Figure 2, *t*_(144)_ = −1.34, *p* = 0.2525; *t*_(344)_ = −6.55, *p* = 0.0028; *t*_(544)_ = −12.44, *p* = 0.0002; *t*_(744)_ = −21.67, *p* < 0.0001; *t*_(944)_ = −20.34, *p* < 0.0001). Moreover, with an increase in the length of the promoter sequence, we detected a corresponding gradual enhancement of reporter plasmid activity, with the pGL3-basic-*AhHsp70*p-944 plasmid showing the highest activity (Figure 2; *t*_(944, 744)_ = −10.98, *p* = 0.0005; *t*_(944)_ = −20.34, *p* < 0.0001). Together, these results indicate that the sequence from −744 bp to 0 bp may represent the basal promoter region, whereas that from −944 bp to −744 bp constitutes the core regulatory region.

### 2.3. Characterization of the Interaction between Transcription Factor AhHsf and AhHsp70p

**Figure 3:**
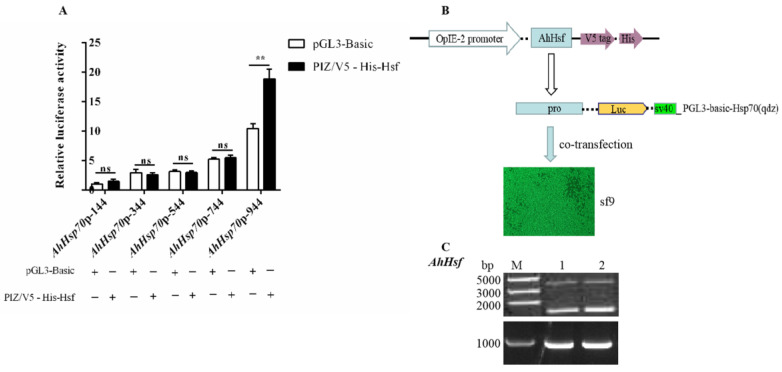
Overexpressed *AhHsf* enhanced the activity of *AhHsp70*p in vitro. In this experiment, *Spodoptera frugiperda* (Sf9) cells were used for the stable expression of AhHsp70 in vitro. (**A**) Characterization of the interaction between transcription factor *AhHsf* and *AhHsp70*p. Analysis of the luciferase activity of *AhHsp70*p in response to the overexpression of *AhHsf* based on a dual-luciferase reporter assay system. pGL3-Basic was used as a control. (**B**) Schematic diagram of Sf9 cells co-transfected with *AhHsf* and *AhHsp70*p luciferase reporter plasmids. (**C**) Agarose gel electrophoresis of the *AhHsf* sequence and double-enzyme digestion of the recombinant plasmid. M denotes a Trans DNA marker and lanes 1 and 2 show samples from duplicate analyses. All values are shown as the mean ± SD. The data were analyzed using the Student’s *t*-test. ** *p* < 0.01, extremely significant; ns, not significant.

Sf9 cells were co-transfected with an *AhHsf* overexpression vector (PIZ/V5-His-Hsf) (Figure 3C and Supplementary Figure S3F) and *AhHsp70*p expression vector, and a DLR assay was used to determine the influence of *AhHsf* overexpression on the activity of the target gene promoter *AhHsp70*p (Figure 3B). The DLR assay results revealed that compared to cells transfected with the control vector, *AhHsf* overexpression significantly enhanced the activity of *AhHsp70*p in vitro (Figure 3A, *t*_(144)_ = −1.82, *p* = 0.1435; *t*_(344)_ = 1.1, *p* = 0.3335; *t*_(544)_ = 0.79, *p* = 0.4754; *t*_(744)_ = −0.81, *p* = 0.4633; *t*_(944)_ = −7.94, *p* = 0.0014).

### 2.4. Expression Levels of AhHsp70 Following In Vitro Transfection

**Figure 4:**
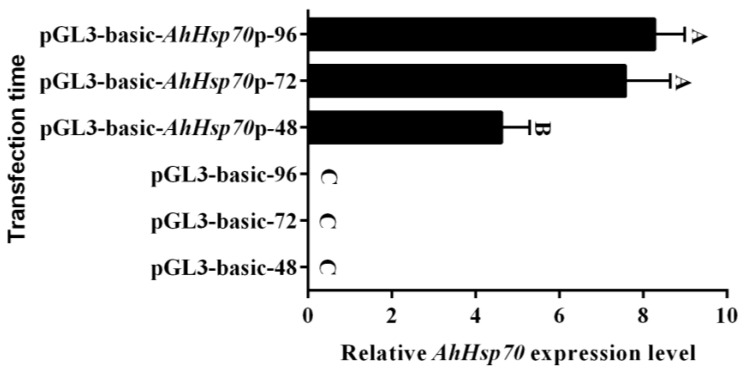
Expression analysis of *AhHsp70* gene at 48, 72, and 96 h after transfection in *Spodoptera frugiperda* *(*Sf9) cells. Relative mRNA levels were determined using the 2^−ΔΔCt^ method and normalized to those of the *β-actin*. The figure shows data on the relative *AhHsp70* gene expression levels analyzed using one-way ANOVA followed by the least significant difference (LSD) test and bars with different letters indicate significant differences (*p* < 0.05). All values are shown as the mean ± SD of three replicates and pGL3-Basic was used as a control.

The level of *AhHsp70* mRNA expression following in vitro transfection was determined by RT-qPCRThe results indicated that the levels of *AhHsp70* expression increased with the extension of Sf9 cell proliferation time from 48 to 96 h (*F*_(__5,12__)_ = 128.51, *p* < 0.0001), although there was no significant difference between the expression levels at 72 hand 96 h (Figure 4, *F* = 0.86, *p* = 0.4067).

### 2.5. Transcription Factor AhHsf Upregulates the Expression of AhHsp70 In Vitro

**Figure 5:**
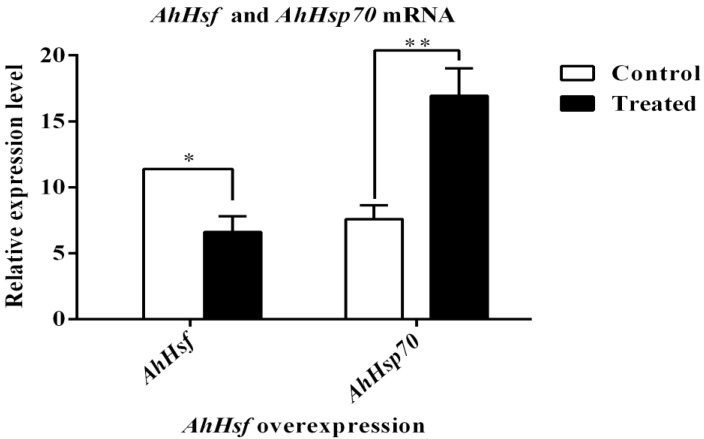
Effect of *AhHsf* overexpression on the level of *AhHsp70* expression. Relative mRNA levels were determined using the 2^−ΔΔCt^ method and normalized to those of the *β-actin* gene. The data were analyzed using the Student’s *t*-test. * *p* < 0.05, significant; ** *p* < 0.01, extremely significant; ns, not significant. All values are shown as the mean ± SD of three replicates and pGL3-Basic was used as a control.

The expression of *AhHsp70* and *AhHsf* in co-transfected cells was determined using RT-qPCR, and the results indicated that there was a significant increase in *AhHsf* and *AhHsp70* expression compared to the control group (Figure 5, *t*_(*AhHsf*)_ = −9.50, *p* = 0.0109; *t*_(*AhHsp70*)_ = −7.16, *p* = 0.0020). These findings indicate that overexpression of *AhHsf* can significantly promote the expression of *AhHsp70* and that this gene plays a regulatory role in the activation of *AhHsp70*.

### 2.6. A Simple Model for Regulation of Transcriptional Gene Expression Following Heat Shock

**Figure 6:**
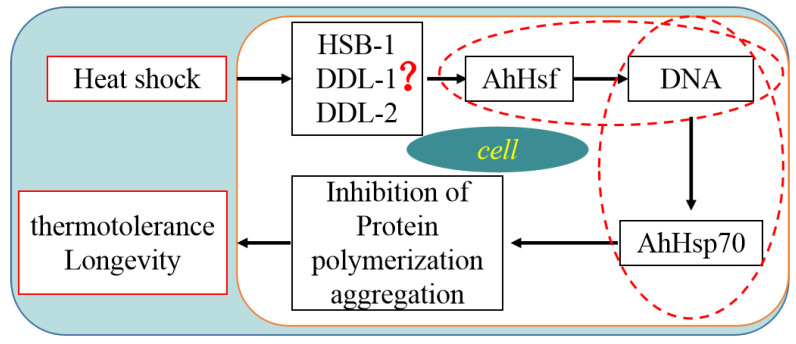
Proposed hypothetical model of the heat shock-induced transcriptional regulation of *AhHsp70* gene expression. (HSB-1: Heat shock factor binding protein 1; DDL: WAS protein family homolog).

Based on the findings of the present study and the Kyoto Encyclopedia of Genes and Genomes pathways, we propose a model illustrating how gene transcription might be regulated following heat shock (Figure 6). In this model, an increase in environmental temperature initiates a signal transduction pathway that activates the transcription factor *AhHsf* and upregulates the expression of *AhHsf* target genes. Subsequently, *AhHsf* activates the expression of its target *AhHsp70* thereby enhancing thermotolerance.

## 3. Discussion

Heat shock factor 1 (Hsf1) plays an essential role in protecting cells from protein-damaging stress associated with protein misfolding [27]. Previous studies have indicated that Hsf’s plays a central role in remodeling the chromatin structure of Hsp promoters via constitutive interactions with a high-affinity binding site, the heat shock element (HSE) [44,45,46,47,48]. By binding to a gene promoter, transcription factors are typically assembled to form “transcriptional switches” that are capable of controlling gene expression [49].

In our previous study, based on RT-PCR and RNAi analyses, we found that the transcription factor *AhHsf* regulates the transcriptional expression of *AhHsp70* and plays a key role in the thermotolerance of *A. hygrophila* [42,43]. In the present study, we isolated and determined the sequence of the promoter region of this gene (*AhHsp70*p) based on I-PCR. Subsequent bioinformatics analysis revealed that the sequence had certain characteristics common to promoter regions, although it appeared to be deficient in CpG islands. In contrast, other studies have found that some promoters contain CpG islands, which are believed to play an important role in the epigenetic regulation of these genes and are generically equipped to influence local chromatin structure and assist in the regulation of gene activity [50,51,52,53]. Moreover, promoters serve as key *cis*-acting elements that regulate gene expression, and it is generally believed that the *cis-* and *trans*-regulatory machinery in promoter regions are basic requirements for gene expression [54]. In 2020, Jia et al. identified six *AhHsp70*s (*hsp70-1*, *hsp70-2*, *hsp70-3*, *hsp70-4*, *hsp70-5*, *hsp70-6*) in *A. hygrophila* [55]; all six Hsp70s of *A. hygrophila* had a non-organellar consensus motif RARFEEL [56], and the C-terminal sequences included the EEVD motif for cytoplasmic localization.

Promoter deletion analysis is one of the primary and most widely used techniques employed to determine whether they are *cis*-acting elements or specific transcription factor binding sites within a promoter that are primarily responsible for the transcriptional regulation of a particular gene [49,57]. Luciferase (firefly and *Renilla* luciferases) genes have been used extensively as reporter genes because of their sensitivity and efficiency [18,54,58]. For example, Apriana et al. demonstrated the root-specific expression of an alkenal reductase gene (*OsAER1*) in *Oryza sativa*, based on the deletion analysis of the *OsAER1* gene promoter [59]. Through deletion analysis in transgenic rice, Chen et al. reported that the *OsHAK1* promoter (Dp3037 sequence) a potentially suitable candidate for regulating the expression of osmotic/drought stress-responsive transgenes [60]. Similarly, Yao et al. used deletion analysis to identify a 22-bp DNA *cis*-element in the *SPHK1* promoter that plays an essential role in transcriptional activation [57]. In the present study, we used the same analytical approach and our results indicated that *cis*-acting elements controlling *AhHsp70* transcriptional expression in response to heat stress are located within a promoter region between base pairs −944 and −744. However, to facilitate more accurate localization of these *cis*-element positions, we will need to conduct further deletion analysis that entails progressively finer truncations.

To elucidate the mechanisms by which the heat resistance in *A. hygrophila* is regulated, we previously identified a heat shock factor *(AhHsf*) and its downstream target gene *AhHsp70* and established that they play important roles in the thermotolerance of *A. hygrophila* [43]. In the current study, we further demonstrated that transcription factor (*AhHsf*) binding to the *AhHsp70*p sequence (from −944 bp to −744 bp) activated its transcriptional expression in vitro based on promoter deletion, the DLR system, and RT-qPCR analyses. Collectively, the in vitro results obtained in the present study and in vivo results obtained from previous work [43] provide convincing evidence that *AhHsf* binds to *AhHsp70*p to activate the AhHSF–AhHSP signaling pathway, thereby promoting transcriptional expression of *AhHsp70*, which in turn contributes to the enhancement of heat tolerance in *A. hygrophila.* These findings are similar to those reported in *Drosophila melanogaster* and yeast, in which cooperative interactions between Hsf’s and its target binding sites (promoter and heat shock elements) regulate the transcriptional expression of heat shock protein genes [26,48,61]. Accordingly, we inferred that *AhHsf* acts directly on the *AhHsp70* gene promoter to induce the transcriptional expression of *AhHsp70*, thereby enhancing the tolerance of *A. hygrophila* to high environmental temperatures. Nevertheless, we have established that overexpressed *AhHsf* can promote *AhHsp70* gene expression at the transcriptional level, and further analyses will be needed to confirm whether our observations can be replicated at the protein level.

## 4. Materials and Methods

### 4.1. Experimental Insects and Host Plants

Adult *A. hygrophila* were collected in July 2018 from an alligator weed covered pond in Changsha (28°11′49″ N, 112°58′42″ E), Hunan Province, China, using a sweeping method. These specimens were maintained on alligator weed plants in a laboratory at the Institute of Plant Protection, Chinese Academy of Agricultural Sciences, Beijing, and the Langfang Experimental Station of the Chinese Academy of Agricultural Sciences, Hebei Province, under controlled conditions (temperature, 26 ± 2 °C; relative humidity, 75% ± 5%; photoperiod, 12:12 h light:dark regime) [62]. To eliminate maternal effects, the flea beetles were cultivated for three generations before commencing the experiments. Groups of five females and five males were each placed in circular containers of 8 cm diameter and 12 cm height containing fresh *A. philoxeroides* stems. The gender of the experimental insects was determined based on the presence of a groove at the end of the abdomen, which is present in males but absent in females [37].

Roots of *A. philoxeroides* were collected from standing water at the Institute of Plant Protection, Hunan Academy of Agricultural Sciences, and planted in plastic boxes (25 × 25 × 20 cm) containing sterilized soil. The plants were subsequently grown in a greenhouse at the Langfang Experimental Station, with daily watering.

### 4.2. Expression Vector and Cell Lines

In the DLR assay system (Transgen, Beijing, China) thea firefly (*Photinus pyralis*) luciferase gene vector pGL3-basic (Promega, Madison, WI, USA) was used as a reporter plasmid, with the *Renilla* (*Renilla reniformi*) luciferase gene vector pRL-TK (Promega) as an internal control plasmid. The plasmids were kindly donated by Professor Ganqiu Lan of the College of Animal Science and Technology, Guangxi University. One element of the pIZ/V5-His vector (catalog number: V8000-01, Invitrogen, Carlsbad, CA, USA), an insect overexpression vector used for the transcription factor, was purchased from Invitrogen, and the other was kindly donated by Professor Changyou Li of the Laboratory of Biological Control for Insect Pests, Center for Advanced Invertebrate Cell Culture and Cell Engineering, Qingdao Agricultural University. All vector sequences were confirmed by Sangon Biotech Co. Ltd. (Shanghai, China). Sf9, donated by Professor Changyou Li, were cultured in TNM-FH insect medium (Hyclone, Gibco, New York, NY, USA) supplemented with 10% fetal bovine serum (Gibco, New York, NY, USA) in humidified air containing 5% CO_2_ at 27 °C in a biochemical incubator (BPC-250F; Yiheng, China). These cells were sub-cultured at 3- to 5-day intervals and used for transfection experiments when cell densities reached approximately 80–85% confluence (at approximately five days) (Supplementary Figure S4).

### 4.3. Sample Collection and In Vitro Experiments

To determine the *AhHsp70*p sequence via reverse PCR, each group comprising five pairs of newly emerged (<12 h following eclosion) *A. hygrophil*a adults was placed together in a circular container (12 cm in height and 8 cm in diameter) provisioned with fresh alligator weed leaves. The beetles were exposed daily to a temperature of 33 °C for a 4-h period (10:00 to 14:00) in a constant temperature incubator (RPX-450; Colin, Beijing, China). The sampled individuals were immediately frozen in liquid nitrogen for DNA or RNA extraction, or stored in a −80 °C freezer (DW-86L628; Haier, Tsingtao) until further analyses.

For the DLR assays, vectors with different *AhHsp70*p promoter lengths (pGL3-basic-*AhHsp70*p-144, pGL3-basic-*AhHsp70*p-344, pGL3-basic-*AhHsp70*p-544, pGL3-basic-*AhHsp70*p-744, and pGL3-basic-*AhHsp70*p-944) were used and the vector pIZ/V5-His-AhHsf was used to overexpress the transcription factor *AhHsf*. After transfection of the dual-luciferase reporter vector into Sf9 cells, the activity of *AhHsp70*p was analyzed using the DLR system, and the level of *AhHsp70* expression was determined using qPCR. After co-transforming Sf9 cells with pIZ/V5-His-AhHsf and the *AhHsp70*p dual-luciferase reporter gene vector, the *AhHsp70*p activity was analyzed compared with that of a control group lacking the transcription factor overexpression vector, and the levels of *AhHsp70* and *AhHsf* expression were determined based on RT-qPCR.

### 4.4. DNA or RNA Extraction and Reverse PCR

*Agasicles hygrophila* genomic DNA was extracted using the phenol–chloroform method and total RNA was extracted using TRIzol (Invitrogen) in accordance with the manufacturer’s instructions [63]. The isolated total RNA was either stored at −80 °C for further use or converted to first-strand cDNA, synthesized using a reverse transcription kit (TransScript^®^ All-in-One First-Strand cDNA Synthesis SuperMix for qPCR [One-Step gDNA Removal], AT341-02, TransGen Biotech, China) for subsequent RT-qPCR. The isolated DNA was used to determine the *AhHsp70*p sequence by I-PCR (Supplementary Figure S5). To characterize the promoter by PCR, primers were designed using Primer 5.0 (Table 1), and the products were cloned into a pEASY-T3 vector (TransGen, Beijing, China) and then sequenced.

### 4.5. Relative Quantitative Real-Time PCR

The *AhHsp70* and *AhHsf* expression levels were assessed via RT-qPCR using a TransStart Green qPCR SuperMix Kit (AQ141-04-p, Transgen, Beijing, China) and an ABI Prism 7500 Real Time PCR System (Applied Biosystems, New York, NY, USA). All PCR reactions were performed in triplicate using the primers listed in Table 1. Reactions were performed as 20 µL reaction mixtures comprising 10 µL of 2× TransStart^R^ Tip Green qPCR SuperMix, 0.4 µL of Passive Reference Dye II, 0.4 µL of forward and reverse specific primers, 1 µL of cDNA template, and 7.8 µL of ddH_2_O. *β-actin* was used as an internal reference standard and relative expression levels were determined using the 2^−ΔΔCt^ method with the following formula:

^ΔΔ^Ct = (Cp _target_ − Cp _reference_)_treatment_ − (Cp _target_ − Cp _reference_)_control_

### 4.6. AhHsp70p Sequence Analysis

We used the Berkeley Drosophila Genome Project neural network promoter prediction (http://www.fruitfly.org/seq_tools/promoter.html/, accessed on 24 February 2021) to predict the 5′ end transcription start site of *AhHsp70* gene, and *AhHsp70*p binding sites were predicted using JASPAR, a database of transcription factor binding profiles (http://jaspar.genereg.net/, accessed on 24 February 2021) and TargetScan (http://www.targetscan.org/mamm_31/, accessed on 24 February 2021). CpG islands in *AhHsp70*p were determined using the Methprimer online software (http://www.urogene.org /methprimer/, accessed on 26 February 2021).

### 4.7. Synthesis of AhHsp70p Insertion Fragments

To generate an *AhHsp70*p target luciferase reporter, we designed five deletion *AhHsp70*p specific primers (Table 1) using the CE V1.04 software. A *Kpn*I restriction site sequence with approximately 15–25 bp homologous to the vector region was added to the 5ʹ end of the forward primer and an *Xho*I restriction site with approximately 15–25 bp homologous to the vector was added to the 5ʹ end of the reverse primer. The target fragment was obtained by PCR amplification using genomic DNA as a template. PCR amplification reactions were performed using a total reaction volume of 25 µL comprising 2.5 µL of 10× PCR buffer (10 µM), 0.5 µL of dNTPs (2.5 mM), 0.5 µL of *Taq* DNA polymerase (TransGen Biotech, China), 1 µL of each gene-specific primer pair, 0.5 µL of genomic DNA template, and 19 µL ddH_2_O. The PCR products were purified using an AxyPrep^TM^ DNA Gel Extraction Kit (Axygen) and cloned into a pEASY-T3 vector (TransGen, Beijing, China). The veracity of the plasmids was confirmed using commercial sequencing (Sangon Biotech, Shanghai, China).

### 4.8. Construction of Luciferase Reporter Plasmids and AhHsp70p Luciferase Activity Assays

The target plasmids described in Section 4.5 were digested using *Kpn*I and *Xho*I, and the resulting PCR products were subcloned into the *Kpn*I and *Xho*I sites of the luciferase reporter vector pGL3-basic using a homologous one-step cloning kit (*Trelief^TM^* SoSoo Cloning Kit, TSV-S2; TsingKe Biotech, Shangai, China) according to the manufacturer’s instructions, with pRL-TK being used as a control vector. Digestion reactions were performed using a total reaction volume of 50 µL containing 1 µg of pGL3-basic vector or insert fragment plasmids, 5 µL of 10× Cutsmart buffer (NEB), 1 µL of *Kpn*I-HF, and 1 µL of *Xho*I, made up to the final volume with ddH_2_O. Following activation, the mixture was incubated for 4 h at 37 °C, after which *Xho*I was inactivated at 65 °C for 20 min, and 5 µL 10× gel loading dye was added to inactivate *Kpn*I-HF. Recombinant DNA was transformed into *Escherichia coli* DH5α cells (TsingKe), and plasmids were sequenced (Sangon Biotech, Shanghai, China).

Sf9 cells were co-transfected with 1.5 µg luciferase recombinant reporter plasmids (pGL3-basic-*AhHsp70*p-144, pGL3-basic-*AhHsp70*p-344, pGL3-basic-*AhHsp70*p-544, pGL3-basic-*AhHsp70*p-744, and pGL3-basic-*AhHsp70*p-944) (Supplementary Figures S3A,E and S6) and 150 ng of the pRL-TK internal control plasmid using Cellfectine^®^ II Reagent (Invitrogen). At 48, 72, and 96 h post-transfection, cells were lysed, and the luminescence of firefly and *Renilla* luciferases was determined using a Multi-Mode Microplate Reader (Infinite M Plex; Tecan, SWIT) and a *TransDetect*^®^ double-luciferase reporter assay system (FR201, TransGen Biotech, China), which enhanced the experimental accuracy, in accordance with the manufacturer’s protocol. *AhHsp70* expression levels were determined using qPCR.

### 4.9. Assays for the Interaction between Transcription Factor AhHsf and AhHsp70p

To determine whether the transcription factor *AhHsf* directly regulates the expression of *AhHsp70*p in vitro, we constructed the overexpression vector pIZ/V5-His-AhHsf harboring *AhHsf* and co-transfected this into sf9 cells with *AhHsp70*p reporter plasmids. After co-transfection, *AhHsp70*p-related luciferase activity was measured using the DLR system following the manufacturer’s protocol, and *AhHsp70* and *AhHsf* expression levels were determined based on RT-qPCR analysis.

### 4.10. Statistical Analysis

Statistical analyses were performed using SAS software v8 for Microsoft Windows and GraphPad Prism software (version 6.0; GraphPad Software Inc., San Diego, CA, USA). One-way analysis of variance (ANOVA; SAS Institute Inc., USA) was used to analyze the differences among treatments, followed by a least significant difference (LSD) test for multiple comparisons. *AhHsp70* and *AhHsf g*ene expression levels and *AhHsp70*p relative luciferase activity after co-transfection of cells with the *AhHsf* overexpression and *AhHsp70*p reporter plasmids were analyzed using the Student’s *t*-test. The results are presented as mean values ± standard deviation (SD); *p*-values of 0.05 or lower were considered significant (* *p* < 0.05; ** *p* < 0.01).

## 5. Conclusions

In summary, we isolated *AhHsp70*p from *A. hygrophila,* determined its sequence using I-PCR, and characterized its composition based on bioinformatics analysis. We subsequently examined the activity of *AhHsp70*p via cell transfection and promoter deletion analyses, and systematically studied the interaction between co-transfected *AhHsf* and *AhHsp70*p using a dual-luciferase reporter assay system, with the levels of *AhHsp70* and *AhHsf* expression in co-transfected cells being determined based on RT-qPCR analysis. Our findings indicated that the upstream sequence of the *AhHsp70* promoter may contain core functional regions between base pairs −944 and −744. The DLR assay system results revealed that overexpression of *AhHsf* could significantly enhance the activity of *AhHsp70*p, and qPCR assays indicated that the level of *AhHsp70* expression increased with the extension of Sf9 cell proliferation time within a certain limit, which is conceivably attributable to the fact that Sf9 cells cease proliferating after reaching a certain density or that cells are subject to a certain extent of apoptosis. Moreover, we found that *AhHsf* overexpression significantly enhanced the expression of *AhHsp70* in transfected cells. Collectively, the findings of this study enabled a preliminary characterization of upstream regulatory mechanisms underlying the transcriptional regulation of *AhHsp70* expression, which entailed binding of the transcription factor *AhHsf* to upstream *cis*-acting elements (promoter region from −944 bp to −744bp) of *AhHsp70* to activate the AhHSF–AhHSP signaling pathway at the transcriptional level, thereby enhancing the transcriptional expression of *AhHsp70* to protect *A. hygrophila* from high-temperature damage.

## Figures and Tables

**Table 1:** Sequences of oligonucleotide primers used in this study, designed using CE V1.04 and primer 5.0 software. Note: the underline sequences are cleavage sites of restriction enzyme and the bases underlined with a wavy line are the homologous arm sequence of the upstream terminal of the vector.

Primers Name	Sequence (5′-3′)	Application	Enzyme
1279F	CAGACATTTACAACATACGCAG	Inverse PCR	*Hind*III
**305R**	TTTGGCTTACCACTCACG	Inverse PCR	
51F	CGCAATCTAAGAACAAACC	Sequence verification	
1106R	CAGCATCACCAAGAAGGC	Sequence verification	
−944F	atttctctatcgataggtaccAAAGTCACAAATGAATGCAGTTATTTAT	Cloning	*Kpn*I
−944R	acttagatcgcagatctcgagTATTTTCCAAGTTTAATACTTCACAAATATATT	Cloning	*Xho*I
−744R	acttagatcgcagatctcgagATTCTCGGCGTAAATCGAAAAG	Cloning	*Xho*I
−544R	acttagatcgcagatctcgagAACTTCAAGAAAACAAATTGAATTTCC	Cloning	*Xho*I
−344R	acttagatcgcagatctcgagGGCACCGATCAGATCATGTTTT	Cloning	*Xho*I
−144R	acttagatcgcagatctcgagCTTTCATCCTGCTATCCAATATATCAT	Cloning	*Xho*I
q-AhHsp70-F	GCCACAGCTGGTGACACACA TCT	RT-qPCR	
q-AhHsp70-R	AGCTCTTTCGGCAGCAGTCC	RT-qPCR	
Q-AhHsp70-F	GGTAGCAATGAATCCCAG	RT-qPCR	
Q-AhHsp70-R	TTACCTTGGTCGTTGGCA	RT-qPCR	
Q-Hsf-F	TGCCAACGACCAAGGTAA	RT-qPCR	
Q-Hsf-R	ACACACCCACACAGGAATA	RT-qPCR	
*β*-actin-F	GGAATGGAAGCCTGTGGTATC	RT-qPCR	
*β*-actin-R	CATTCTGTCGGCAATACCTGG	RT-qPCR	

## Data Availability

The data presented in this study are available on request from the corresponding author.

## Supplementary Materials

The following supporting information can be downloaded at: https://www.mdpi.com/article/10.3390/ijms23063210/s1.
