# Supplementary material for: Regulatory Mechanism of Transcription Factor AhHsf Modulates AhHsp70 Transcriptional Expression Enhancing Heat Tolerance in Agasicles hygrophila (Coleoptera: Chrysomelidae)
Source: Int J Mol Sci. 2022 Mar 16;23(6):3210. doi: 10.3390/ijms23063210 (PMC8955217; doi:10.3390/ijms23063210)
Supplement: Supplementary file 1 [file ijms-23-03210-s001.zip › ijms-1611486-supplementary.pdf]

Supplementary Materials:

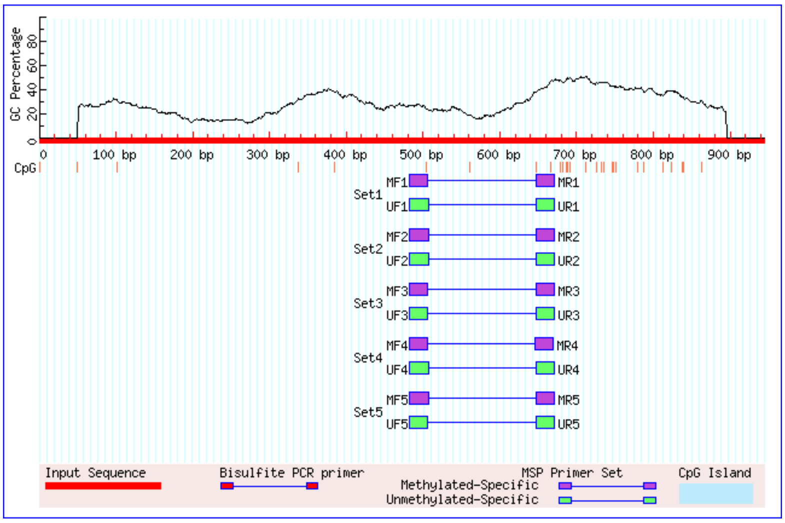

Supplementary figure S1. CpG island prediction of *AhHsp70* gene promoter by Methprimer software of *A. hygrophila.* Methprimer analysis showed that the *AhHsp70*p did not contain CpG island.

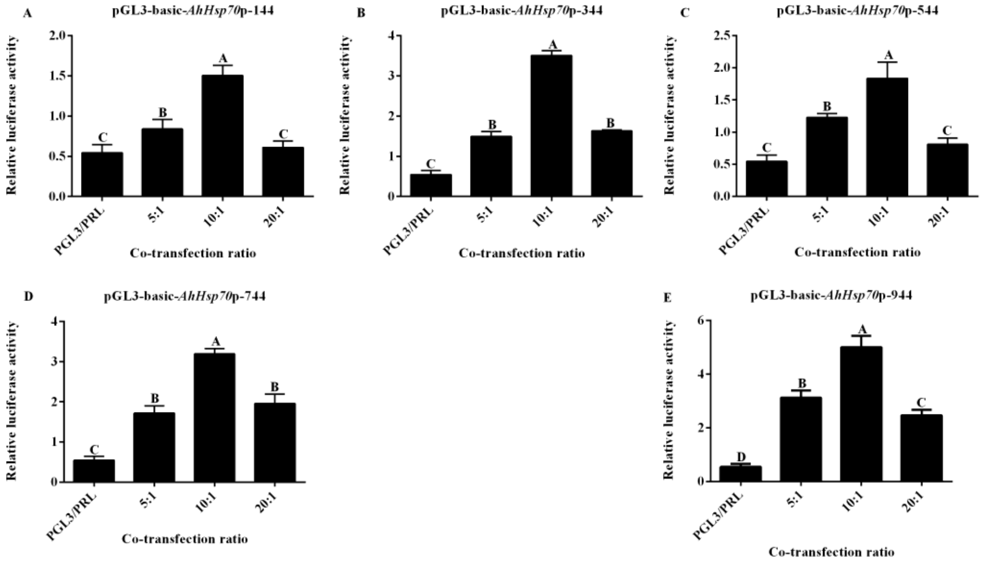

Supplementary figure S2. Standardization of the co-transfection ratio for recombinant and control plasmids (A-E) and the luciferase activity of *AhHsp70*p. The data were analyzed using one-way ANOVA followed by the least significant difference (LSD) test (p values < 0.05). All values are shown as the means ± SD of three repeat measurements and bars with different letters indicate significant differences (p < 0.05).

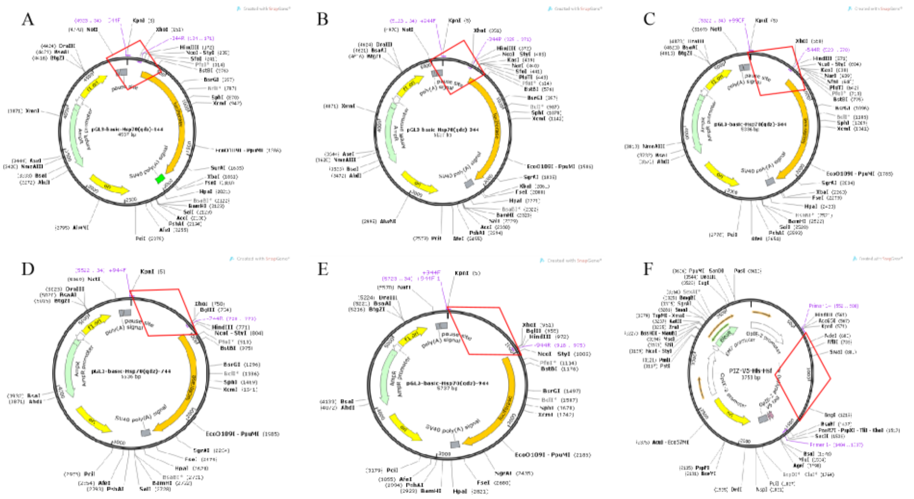

Supplementary figure S3. Construction of recombinant reporter plasmids with promoter deletion fragments and luciferase reporter gene vector, and PIZ/ V5-His-HSF overexpression vector for AhHsf. A, pGL3-basic-*AhHsp70*p-144; B, pGL3-basic-*AhHsp70*p-344; C, pGL3-basic-*AhHsp70*p-544; D, pGL3-basic-*AhHsp70*p-744; E, pGL3-basic-*AhHsp70*p-144; F, PIZ/V5 - His-Hsf.

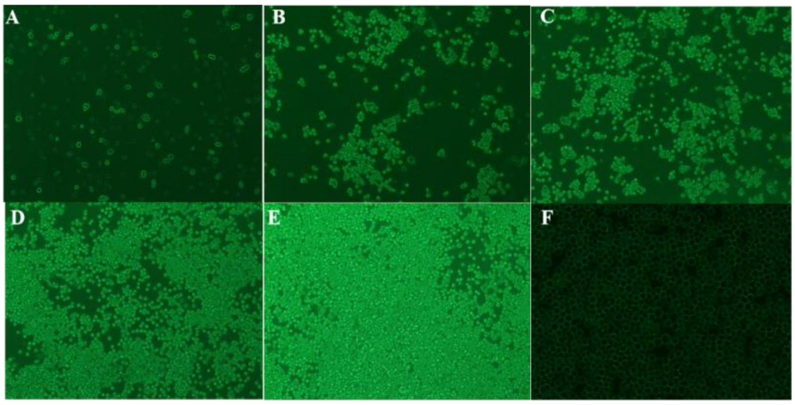

Supplementary figure S4. Sf9 cell lines culture atatus before transfection. A, culture sf9 cells for one day; B, culture sf9 cells for two days; C, culture sf9 cells for three days; D, culture sf9 cells for four days; E, culture sf9 cells for five days; F, culture sf9 cells for six days.

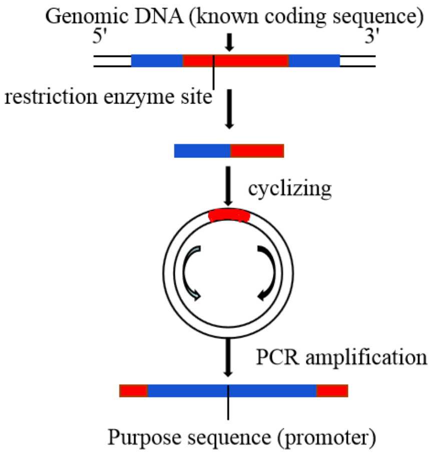

Supplementary figure S5. Schematic diagram of Inverse - PCR

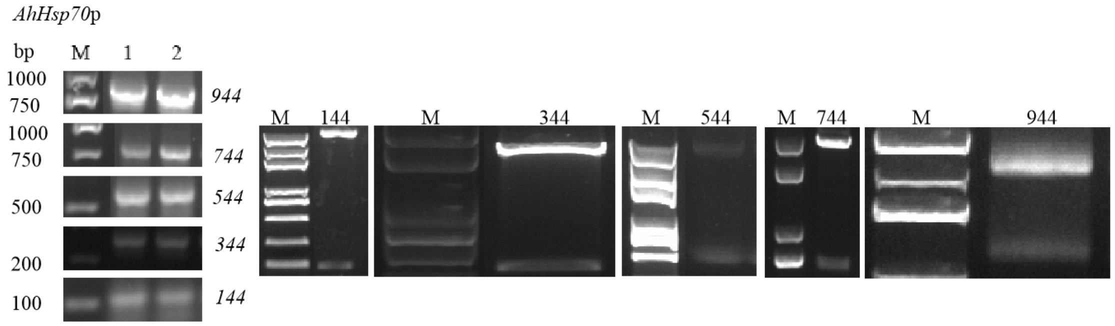

Supplementary figure S6. Agarose gel electrophoresis of deleted *AhHsp70*p sequences and double-enzyme digestion of recombinant plasmids.

Supplementary Table S1: The number of days in different temperature ranges and annual maximum temperature range in summer of Changsha city from 2003 to 2013. (China Meteorological Data, http://data.cma.gov.cn/).

Month	frequencies of the highest daily temperature in different temperature ranges			Annual maximum temperature range
	< 33.0°C	33.0～36.0°C	> 36.0°C	
**May**	85.63%	14.08%	0.29%	30.0~35.7°C
**June**	68.6%	28.1%	3.3%	35.0~37.6°C
**July**	21.4%	36.1%	42.5%	37.0~39.7°C
**August**	40.2%	28.7%	32.1%	37.2~40.6°C
**September**	76.7%	18.2%	5.1%	34.2~37.4°C
